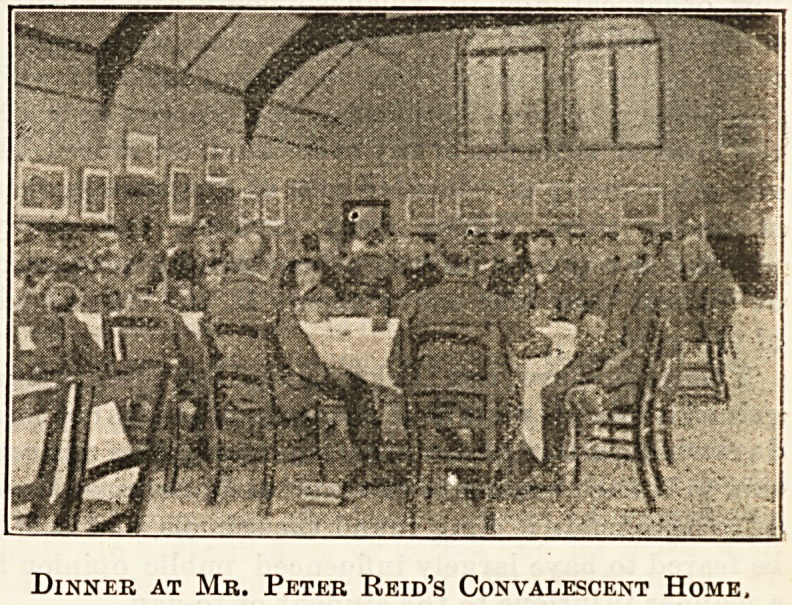# The Hospital.—Christmas Appeal Supplement

**Published:** 1895-12-14

**Authors:** 


H.R.H. THE PRINCE OF WALES.

Albert Edward P.
1895.

Patron of the Royal National Pension Fund for Nurses.

### CHRISTMAS APPEAL SUPPLEMENT—ILLUSTRATED.

### H.R.H. the Prince of Wales.

PATRON OF THE ROYAL NATIONAL PENSION FUND FOR NURSES.

AFTER the publication of the portrait of H.R.H. the Princess of Wales, which appeared in THE HOSPITAL last Christmas, we received numerous requests not only from this country, but from India and the Colonies, urging that we should this year, if possible, publish a portrait of the Prince to accompany that of the Princess. As one nurse working in a distant part of India wrote: "The Princess looks lonely without her husband, and it would be most gratifying to me to have the Prince's portrait and autograph to hang in my room beside that of his wife." When we communicated these facts to H.R.H. he most graciously and promptly sent the excellent photograph we reproduce to-day, to which he at the same time attached his autograph. Members of the Pension Fund, nurses of high and low degree and hosts of other readers of THE HOSPITAL, are now enabled to possess an original portrait of H.R.H. the Prince of Wales, worthy of preservation, which we have no doubt they will prize at its true value, having regard especially to the circumstances under which it is placed in their hands.

### SOME ATTRIBUTES OF THE PRINCE.

It will be in the remembrance of the thousands of nurses who have had the privilege of being present at the Pension Fund gatherings at Marlborough House, that the Prince, together with every member of his family and household, has always exerted himself to make the visitors feel welcome and at home. He is unequalled as a host, ready, considerate, kindly and most gracious to all. Indeed, those who know him best never tire of declaring that he is one of the kindest and most considerate of men. The Prince's affability, his intimate knowledge of the relative positions of individuals, and the precedence due to such positions, are as remarkable as they are unique. On one occasion, we remember, at a military luncheon, where there were some twelve monarchs and princes of the blood of various nations present, with many other illustrious personages, when the order of precedence was found most difficult to settle, an appeal was made by the General in command to the Prince, who in three minutes arranged everybody in his proper order without any hesitation or apparent difficulty. This incident reveals the power of acquiring knowledge which the Prince possesses to a remarkable degree. He never spares himself any labour, and will take an infinity of trouble to fit himself to undertake each and every duty, so as to discharge it efficiently and thoroughly. The character of the speeches, and especially of the extempore speeches, which we are glad to note the Prince is now making with increasing frequency, exhibits this attribute with convincing clearness. It is popularly supposed that speeches of this kind are usually composed by others. This is not however, the case, and we believe the Prince has never permitted any words to be put in his mouth, from his earliest public appearance to the present time, which had not first of all been weighed and largely shaped by himself. He never forgets a name or a face, and seldom a date, being able to give the exact year, and usually the month and day, of most events in his life. As a son, he has shown a most anxious desire on all occasions to lighten, as far as possible, the onerous duties which devolve upon his Sovereign mother the Queen, and it is divulging no secrets to say that his great tactfulness has been widely recognised and appreciated, not only by members of the Royal Family, but by statesmen of all parties and every shade of political opinion.

### AT HOME.

H.R.H. is most orderly and methodical in all his arrangements. Nothing is ever allowed to be untidy in his room or about his person. Out of doors a dead tree or branch unremoved, a hedge unclipped, or a weedy walk are a positive misery to him. Like the Princess of Wales, he loves animals, chiefly dogs, and always has one special favourite, who goes everywhere with him and sleeps in his room. Ever since the Duke of Clarence's death his great pet has been his late son's dog Venus, who never leaves him. He takes a keen interest in the news of the day, and has a wonderful knack of getting through the day's newspapers quickly and of digesting all the news. The Prince sets the nation an example as a correspondent. Every letter received is replied to at once, and important correspondence is never neglected for other matters. He writes, too, very quickly, and expresses himself well, having, in fact, a genius for letter-writing. As a husband and father he is thoughtfulness personified; his affection for his children and their affection for him have been illustrated in the most touching way on many occasions. He is a devoted father, especially as regards the Duke of York, who is more like a brother or comrade than a son, and the affection between these two is complete. As a master he is beloved by all who work under him, and the more they come in contact with His Royal Highness the more attached are they to his person by affection and regard. He is very particular as to details, but is most kind and just, and when in the wrong himself he has been heard to apologise to a servant at once.

### His Hold on the Nation.

Often as the Prince has been criticised, we make bold to think and to say that his hold upon the affections and esteem of the people is as remarkable as it augurs well for the future of England. People of our day will not cease to remember the great service which he rendered to the nation by accompanying the Princess of Wales to Russia when Alexander III. lay dying at Livadia and her sister was experiencing the anguish of approaching bereavement. The Prince and Princess arrived too late to see the late Tzar alive, but their arrival brought solace and peace to the Imperial Russian family which no one else could have rendered in anything like the same degree, if at all. As an eloquent writer has well said: "In the long and almost terrible pilgrimage which followed, when the corpse of the dead Tzar was carried in solemn state from the shores of the Black Sea to the tomb in the cathedral that stands on the frozen Neva, the Prince was always at the right hand of the Tzar. Alike in public or in private the uncle and the nephew stood side by side." Writing of this incident, a correspondent of a Parisian paper said: "No other Prince in the world, perhaps, likes his ease better than the heir to the English throne, yet see the terrible task undertaken by him for more than a fortnight, from Livadia to the day of the funeral of Alexander III., accompanying the Russian family twice a day to the religious ceremonies solemnised before the open coffin of the late Emperor, and after each service mounting the steps of the catafalque behind the Empress and Nicholas II. to kiss the brow of the august deceased. His attitude was not less remarkable in the private circle of the Anitchkoff Palace. There he endeavoured, after each of these sad ceremonies, to effect a consoling reaction against grief, being affectionate towards all, and even going the length of playing with the children. This attitude was certainly deliberate, but who can say that it was not sincere? How could it help being highly appreciated, and how could it help bearing fruit? The Russian Royal family, particularly the Emperor Nicholas and the Empress, are deeply grateful for it. Ties have been formed in these days of mourning, and they have assumed a political character which will perhaps last longer than is imagined, and which, as the first result, have inspired the two countries with a desire to live on friendly terms."

This impartial evidence of the effect which the Prince's conduct throughout that trying ordeal had upon the Russian people and European nations not too favourably disposed towards England is a proof of the value of the services His Royal Highness then rendered, not only to the Imperial Russian family, but to our own nation. It is not too much to say that the example of the Prince of Wales on this occasion impressed upon a multitude of his countrymen the importance of devotion to duty. It further encouraged many a man to persevere against every obstacle under the most trying conditions, because he felt that this exhibition of whole-hearted devotion to duty on the part of the highest in the land must nerve his arm and impel his manhood to bear and forbear, that he might have strength to act up to the high standard which the Heir to the Throne had laid down for himself and successfully maintained. The power and influence of example were never more strikingly proved.

### In Sorrow and Sympathy.

And twice the Prince has been brought very near the hearts of the people of England by affliction more closely touching himself—the first time when he was stricken down with severe illness, an illness all the more dreaded, in that typhoid fever proved fatal to his beloved and honoured father; and secondly when his own child, the Duke of Clarence, was taken away at a moment when a bright future seemed assured him. The Prince of Wales' illness raised universal feelings of sympathy for the Royal mother and for the devoted wife, and of appreciation for one who had ever been ready to encourage and help in the charitable work of the nation. The death of the Duke of Clarence deepened the sympathy and admiration felt for the mourning father. Just as in Russia the Prince followed the bier of the Tzar, in spite of a knowledge of personal danger, so he braved the warnings of his physician when the sad procession left Sandringham, and followed to Wolferton reverentially on foot in spite of the inclement weather which prevailed. He accompanied the remains of his eldest son to Windsor, and on arrival there he continued to follow them on foot to their last resting-place. How fresh these touching incidents are in the memory, and how they went home to the hearts of the mothers and fathers throughout the land. How those hearts warmed to the Prince at that trying time, and were and are animated with the most perfect sympathy for himself and his family. These times of affliction unite those who suffer with those who help to alleviate suffering. The Prince of Wales, in common with those in a less exalted position, has had cause for thankfulness that skilled nursing is ready at hand in times of sickness. Both our Prince and our Princess admire all true followers of so worthy a calling, and in no way could they show this more fully than in their personal interest in the Royal National Pension Fund for Nurses, of which they are respectively not only Patron and President, but kindest and most helpful of friends.

### A Merry Christmas for once at the Hospitals.

The hospitals are distinctly in a Christmas frame of mind this year. Not in the frame of mind of that unhappy paterfamilias who dreads Christmas because it means "bills," but rather in the condition of the schoolboy or undergraduate who sees home in the distance, with all the delights connoted by that dear name at this period of Christmas. One is glad for once to find the moaning and the groaning, the whining and the begging with which we have been so long familiar at Christmas time exchanged for this distinct note of cheerful jubilation. The cause is not far to seek. The sudden up-springing of the Hospital Sunday Fund from a little more than forty to something over sixty thousand pounds, with the "boom" thus given to the hospital question generally in every quarter where the necessities and the invaluable character of hospitals ought to be thoroughly known, has come upon the charitable world like a bright summer day in the midst of the dreariness of our English December. It is a happy augury. May the future of hospitals progressively justify it in every succeeding year.

We think we can claim that hospitals are now known as they never were known before. It has been understood for many years that they did excellent work for the poor and suffering classes; but it was not known before the advent of this journal how vast was that work, how all-important to the industrial breadwinner, how indispensable to the progress of medical science, and how almost more than indispensable, therefore, to the well-being and life of all classes of the community who seek medical aid. Hospitals, in short, during the last ten years have come to be recognised as a distinct department of the public service, and that in spite of the fact that they are mainly supported, not by public money, but by voluntary contributions.

"To him that hath shall be given." The hospitals now have money and public appreciation. Why have they both money and appreciation in these times of fierce money-hunger? For this reason, and for no other, that they have proved to the public that they deserve both the one and the other. Perhaps hospitals always, or at any rate for a very long time, have deserved both the support and the gratitude of the public. But even if they have, they have not always proved their case to the public. This journal, being entirely independent, has turned the light of an independent criticism upon those great charitable institutions, with the result that they and their work are now known to all the world instead of to a mere handful of esoteric supporters, as in the olden times.

There can be no doubt that hospital managers are fully alive to the double necessity, first, of so conducting their institutions as to deserve the approbation of the public, and, secondly, of so placing their facts before the public that there can be no manner of doubt as to their truth and reality. The methods of winning support in the future will not consist, as in the past, of persistent begging, accompanied in some cases by misrepresentation, and in others by what has been little better than downright falsehood. The less reputable among the hospitals have ridden that particular hobby to death and made the more respectable institutions quite ashamed even to look at so sorry a jade. There are indications on all hands that more candid, more independent, and more truly English methods will gradually be established. Among these indications is the renewed vitality which has recently been manifested by The Hospitals Association. That association, under its excellent president, the late Dr. Bristowe, senior physician to St. Thomas's Hospital, rendered much-needed aid and guidance to hospitals at a very critical period; and more especially did it pave the way for improved administration by securing the almost universal adoption of the uniform system of keeping hospital accounts. Dr. Bristowe has been succeeded in the presidency of The Hospitals Association by the Honourable Sydney Holland, one of the most energetic and zealous hospital chairmen whom it has been our fortune to meet with for many a year. The outlook for the future of voluntary hospitals, under the stimulus of Mr. Sydney Holland's activity and that of The Hospitals Association, is of the very brightest.

This brief *résumé* of hospital events and progress during the past year is intended to serve as a kind of Christmas Stocktaking. No doubt a survey of the facts and figures of treatment and cure is more acceptable to some. But it is our object to make our readers familiar with the larger and bolder outlines of hospital life and policy, as well as with the detailed statistics of their daily work. We wish them not only to see the hospitals clearly, but to see them "whole."

On this special Christmas occasion we base our appeal for larger support not only on the past but on the future; not only on what hospitals have done, but on what they are going to do; not merely on proved excellence of work and administration, but on the still higher excellences of administration and work which we have clearly shown them to be aiming at. In a word, at the merry season of Christmas we decline to whine and beg even on behalf of such splendid institutions as our noble modern hospitals. Instead of that we say that, worthy as they have been in the past they are worthier in the present, and hope to be still more worthy of every kind of loyal and devoted support in the future. Let everybody take these plain facts generously to heart, and give them a noble English response; and may God send the hospitals and all of us "A Merry Christmas."

### Choosing a Charity.

Our Christmas supplement is probably unique in this, that the advertisements are, without exception, pertinent to the matters in which readers of The Hospital are interested. It contains the appeals of the various hospitals, stating, in the words of their own officials, their actual wants at the present time. Those who at this season are in doubt where to bestow that charity, which is to an Englishman so essential and necessary a feature of an English Christmas, cannot do better than cast their eyes over the appeals which appear in this week's supplement, and, having done so, to fill in the accompanying *Bankers' Form* in favour of such objects as may most appeal to their sympathy.

### Gay with Flowers.

To those whose limbs are sound, who move about and absorb even unconsciously the sights of a great city, and even more to those whose lives are passed in country amid fields and flowers, it is not always easy to understand how comforting to those in hospital is the sight of flowers and green things. Perhaps it is that they are types of health and beauty; perhaps that they are signs of sympathy and care on the part of those who have provided them; perhaps it it is merely because they fill up a picture which otherwise would be one of dead walls and long distances; but, at any rate, the fact remains that to patients in ordinary, it is wrong. What a relief, then, are the flowers! How their disorderly beauty breaks up the scene, how the bald outlines of the windows are broken by the hanging plants and by the vases of flowers on the sills! For decorative purposes we must have contrast, and, if possible, suggestiveness; and while in the luxuriant country, where all is verdant and redolent of the active growth of Nature, gardeners love to place Italian terraces and marble steps, with pillars and pilasters chiselled with all the art of man, hinting that, beautiful as Nature is, the world is not all wild and uncontrolled—so in the hospital ward, to decorate

A Modern Hospital Ward (St. Thomas's).

all hospitals flowers are a joy. But they must be good ones; they need not be rich and rare, they need not be expensive and out of season, but they must be pure, and sweet, and clean, must be suggestive of perfection and not decay; and often, if of homely character, recalling thoughts of times gone by, they will be the more acceptable.

Look at this picture of a ward in St. Thomas's Hospital! What would it be like without the flowers? The beds with their neat coverlets, each clean and perfect in itself, but wonderfully like to one another; the painted walls, the brightly-polished floor, spotless from end to end, but all the same; the row of chimneys, the row of windows, the row of bed lifts, the symmetrically-arranged gas lamps — everything so orderly and uniform that the patient may well be brought to feel that so far as his form, his temper, or his malady diverges from the is not merely to put in form and colour, but to suggest that the world is not all sickness, that there is a sweet outside, and that there is hope.

To the man of science, however, there are other thoughts which the use of plants and flowers for decorative purposes suggests. He requires that for the sake of his patients the ward shall approach as nearly as possible to the condition of a well-ventilated, light, and airy box, absolutely devoid of everything which can harbour dust or germs, and he looks with small favour upon objects of art as a means of decoration. If they are cheap and common they are bad, while if they are valuable they cannot be either disinfected or thrown away. How different with flowers! They come in fresh and sweet, and before they even shrivel they are thrown away. They cheer the patients and they do no harm, which is more than can be said of every means by which pleasure can be given.

### The Medical Student of To-day.

In one of our illustrations we are introduced to a familiar figure in hospital life, but one which by the general public is always surrounded with a sort of mystery. Stooping over the patient, apparently adjusting a splint or some form of apparatus, is a medical student. How the medical student has been

The Dresser at Work.

misconceived! By some enveloped in a halo of romance, as the devoted seeker after hidden truths, the self-sacrificing benefactor of his race; by others covered with ignominy and ridicule, either as the bloodthirsty sawbones or the beery tippler "walking the hospitals" amid an atmosphere of alcohol and tobacco and silly dissipation; and by others again regarded as the very essence and embodiment of the "good young man." In every case far away from the truth, for, in fact, the medical student of to-day is a curious mixture, and very different from the ideas so commonly entertained about him.

Although the great mass of its recruits enter the ranks of the medical profession from a simple desire to earn a respectable living, there are probably few other callings to which so many are drawn by motives which are only remotely connected with a mere desire for wealth; and so it happens that while the mass of medical students may appear very like the mass of students of any other kind, or, indeed, like any other group of educated men of similar age, there is among them, hidden away perhaps, but always present as a potent leaven, a considerable proportion of earnest men who are students in the truest sense of the word.

Those who have read Dickens and Albert Smith have gained a mental picture of the medical student of their days which, although far away from true, does yet contain in it such elements of truth and of *vraisemblance* as to have made it cling round the schools, and it is to be feared to have largely influenced public opinion in a manner injurious to the student of to-day.

Since the days when even the middle-aged among us were young, the education of a doctor has undergone such changes that those who for years have been absent from the schools, and not been conversant with what has been going on there, can hardly believe, and, in fact, often entirely refuse to admit, that it is possible to turn out practical men by the methods now in vogue. When the writer of this was young, the very day after he entered as a medical student he was turned into the surgery of a large hospital, and, taking his place alongside of an older student, amid the casualties, the cut fingers, the sore legs, and the general *olla podrida* collected in the out-patient dressing-room, was made to pick up what he could, and in what way he could, by practical experience. Practice and the actual treatment of cases—maybe trivial ones, but still patients—began at the beginning. At once the raw and callow youth found himself in the presence of all that was most horrible and at the same time fascinating; at once he found both his interest and his sympathy excited by contact with suffering humanity; and, although many older men will still be found to maintain that by this sudden plunge they gained a something which the modern student does not obtain, yet it must be confessed that this method had its disadvantages from the patients' point of view. The student who was admitted to the wards was but too often a raw youth, who so far as anatomy and physiology was concerned knew nothing, and so far as general knowledge of affairs was concerned knew, if possible, even less.

Things are very different now. However much the evening papers may throw scorn upon the student, and declaim against him his being allowed to be given any responsibility in regard to having charge of patients, it must be remembered that before such a thing is possible, before even the chance of coming in touch with sick people occurs at all, the medical student of to-day is a man of much knowledge, who for two years, at least, has studied the structure of the body and the manner in which its functions are performed. Not only has he passed examinations in general education before entering at all, but for two years he has studied anatomy, physiology, chemistry, and the use of drugs. In all these subjects he has been examined, and until he has passed he has no *locus standi* in the wards. It is no longer the case that the lad fresh from school, without experience or knowledge of the world, is turned loose upon the hospitals, to gain by practice on the patients such knowledge as may carry him through his examinations, and shall guide him in the various exigencies of work in after-life.

Before he can take any office in the wards, the student has gone through a good deal, and he has rubbed off the rawness of his inexperience in the class-room, the dissecting-room, the laboratory, and the lecture theatre, before entering on even the most junior appointment in the wards at all. The modern system has substituted a long period of dull study for the old rush of knowledge and experience which constituted so much of the charm of the early part of the medical student's life, and so far to him the change is a loss. But to the patient it is all gain. The junior student, the dissecting-room lounger, is practically kept out of the wards, and those who are admitted to the serious study of disease have already had their eyes to some extent opened to the more serious side of life.

Much has been said with little knowledge about the injury and annoyance to patients caused by the presence of students in the wards, and by the fact, so readily assumed, that their first and main object is not so much the benefit of the patients as the acquisition of knowledge. It may be true that here and there, at long intervals, a patient may be admitted who from

Taking the Temperature.

nervous irritability or other cause is too easily worried and annoyed by their inquiries and attentions, but it is certain that to the great majority of patients the presence of the stu nts is a blessing and relief.

Among the many pleasant sights to be seen in our hospitals—certainly not among the least so—is the sight of the young people, the students of medicine and the nursing probationers, who are working side by side for the good of the patients; all doing their best, perhaps partly for the sake of each other's approbation, and linked together by their sympathy for the suffering they see around. The constant and unending vigil which is maintained in some of the great hospitals of London is a thing unthought of by the many, but is one which appeals strongly to the imagination. Year in and year out, through all the changes of the seasons, by day and by night, whether we are at business, at pleasure, or in sleep, that watching of the sick goes on, and in the larger hospitals that perpetual readiness for accidents is maintained. Individually, of course, the medical student has his vacations, although often only short ones; but no break is made in the care of the sick. In time of holiday as in time of work, the supply of clerks and dressers must be kept up, and those who read of hospital sports, and clubs, and cricket matches, must not forget that, whatever may be going on in regard to these, the hospitals are never left, and that out of the unpaid labour of the students the service of the sick is being continuously maintained.

One effect of the examination system is to make students work hard, and to much diminish their opportunities for dissipation, unless they would become wastrels altogether. Another is, much to the distress of many parents, to weed out from the student ranks, mostly in the first half of their career, many who are inclined either to idleness or evil courses; and thus it happens that the students who reach the wards are the selected ones out of many who have failed. *Væ victis!* One often wonders, alas! what becomes of those who fail. But of those who survive it may be truly said that London has no reason to be ashamed of that great corps of medical students who do so large a share of the practical work of her hospitals.

### The Dinner Hour.

To the sick as to the healthy the dinner hour is an event of no small importance. But how differently it affects those who are in different stages of recovery! There are patients who, when the time of feeding comes, know well that they depend absolutely and entirely upon their nurse as to the meal they get. Men with fractures of the upper limbs, patients suffering from chronic rheumatism, to whom the food, appetising as it may be, is inaccessible without the help of an assistant; cases of burn who, swathed in cotton wool with just an opening for nose and mouth, can neither see nor feel their way to food, and only by smell and by internal cravings can tell that feeding time has come, are dependent upon the kindly help of nurses and probationers who, sitting beside them with gentle insistance and perhaps with somewhat weary perseverance, spoon in the food. Quite otherwise is it with the convalescents who feed themselves. They, indeed, are people with a past. But the past, illness, accident, or whatever it may have been, is over. They have, as the old woman is reported to have said, "got a new complaint—the convalescence," and all they have to do is to absorb as much fresh air, good food, and sunshine as is possible in the time allowed them by their tickets. Feeding is then the very central point on which convalescence hinges, and, indeed, these convalescent institutions find that in their total of expenses food is no small item. To supply this food, however, they may well apply to the public for increased funds, for they may fairly say that the food consumed is a proper measure of their success. Who does not know how the anxious mother, who takes her convalescent child to the seaside, writes home with pride and thankfulness that her little one is "eating well"? And when the convalescent institutions point to their butcher's bill as their great expense, so far from thinking them extravagant, we must look upon them as succeeding and doing well. Doctors will tell us that in illness they feel the pulse,

Dinner at Mr. Peter Reid's Convalescent Home.

but everyone knows that during convalescence they inquire mostly after the appetite. But appetites are expensive things, as many a poor mother knows, and subscribers to convalescent homes must bear in mind that, however expensive a ravenous appetite may be, it is the best and most hopeful sign of returning health.

### What the Press is Doing for Christmas.

With each succeeding Christmas the number of charitable "funds" initiated in the name of the season has increased until in the present year of grace the sum total contributed by the general public through these channels has reached such proportions as to cause thoughtful people to reflect on the wisdom shown by this nineteenth-century development. But without philosophising, let us glance at the details of some of the Christmas schemes put forward this year by the London Press.

To *Truth* must belong the credit of having been an early pioneer in Christmas charities, having started so long ago as 1880 that wonderful collection of dolls and toys for distribution among the sick little ones in the London hospitals, the exhibition of which has now become an annual institution. How far the elaborate dimensions to which it has at the present time attained are to be attributed to charity, as such, or to other and more personal motives on the part of the contributors and competitors who vie one with the other for the glory of being the sender of the best-dressed doll, it boots not to inquire; the result, at any rate, is the same—real and very great pleasure and so genuine good to the small recipients. This year the show, previous to the distribution, is announced to take place as usual in the Albert Hall on December 18th and 19th. Visitors are admitted free, only being asked to leave their cards at the door. It is hoped to provide 24,000 children with toys from this source for Christmastide, although the money received has as yet fallen short of the amount required to accomplish as much as this. All who know anything of hospitals can testify to the enormous pleasure which this bountiful supply causes amongst the little patients, and the excitement with which the yearly appearance of the *Truth* toys is looked forward to.

The most gigantic scheme of this winter is the fund, originated by "One of the Crowd," *i.e.*, Mr. Greenwood, in the columns of the *Daily Telegraph*, with the object of providing Christmas "hampers" for fifty crippled children. From fifty, in consequence of the ready response, the number rose rapidly to 500, and still the cry was "more," and still contributions rolled in, with the result that the original scheme has grown until the number of cripples to receive hampers has been at last limited to 5,000, in addition to which two hundredweight of coal will also be distributed to each recipient of a hamper, and a further development has arisen in consequence of the quantities of toys of every description which have found their way to Fleet Street; these are being already sent out, carefully packed, to various hospitals and homes, those being selected which are least well known and the more likely to escape general attention. The organisation required to carry out so vast an undertaking is tremendous; but all difficulties have been successfully grappled with by Mr. Richardson, on whose shoulders the responsibility has rested. London has been mapped out into districts, and those parts not covered by existing organisations, such as the Ragged School Union and others, from whom reliable information could be obtained, have been worked by voluntary helpers from special local centres. Volunteers have been numerous, grudging no trouble and sparing no pains, and it is pleasant to hear of the numbers of people of all classes who have come forward to offer their services for this purpose.

But to return to the matter in hand. The *Referee* has for many years past organised each winter a "Free Breakfast and Dinner Fund," founded by "Dagonet," otherwise Mr. G. R. Sims. The beginnings were small and local, the district round the Orange Street Board School being the principal centre, Mrs. E. M. Burgwin, its then head mistress, undertaking the treasurership from the first. Year by year subscriptions have multiplied, last year the sum received amounting to £1,714 odd, the balance-sheet being published in the *Referee* for November 17th last.

The *Daily Chronicle* has organised an appeal for £3,000 to help the Poplar Hospital out of its "terrible dilemma," an example of support to individual charities which we hope may form a precedent for Christmas charity. We are glad to see that funds are pouring in. The *Sun* is asking for contributions in money and kind towards providing destitute children with boots and stockings and warm undergarments. The *Westminster Gazette* is nobly doing its share in the way of "Christmas parties" for the dwellers in the garrets and back rooms of the poorest parts of London. It is true that we have seen no mention as yet of the *Pall Mall Gazette's* Christmas tree of former years, but we hope to see its announcement in due course.

The *Gentlewoman* is offering £200 as Christmas gifts to various charities in Scotland, Ireland, Wales, Yorkshire, and Devonshire.

The cry of "starving children" is one which appeals more strongly than most to the sympathies of those kindly people who have of this world's goods and like to give to those who have not, yet any general system of free feeding for the children of the poor is absolutely untenable from a common-sense point of view. But a plan such as that of the National Food Supply Association, the Memorial Hall, Farringdon Street, E.C., which, working now from six centres in London, supplies cheap, good food for a sum ridiculously small (one penny a dinner), yet covering the cost of everything except the initial expense of the necessary steam plant, is excellent, and should be encouraged in every way. Here is an object upon which the charitable might well spend money in helping to establish such centres, on a self-supporting basis, throughout London. Now however much anyone, on grounds of sober sense, may question the means taken to alleviate the sufferings of the cold and hungry, the contemplation of these many kindly efforts leaves us with at least the glad reflection that there will be few of the inhabitants of this great city this Yuletide who will not have brought home to them in one way or another, something of the meaning of the message of the first Christmas Day—"Peace on Earth, Goodwill towards Men."

### SWEET CHARITY'S GUIDE TO CHRISTMAS GIVERS.

[Our readers are specially requested to use the bankers' form enclosed when sending a contribution to any institution.]

### GENERAL HOSPITALS.

**Charing Cross Hospital,** Agar Street, West Strand.—This institution is situated in the midst of some of the most crowded thoroughfares in the metropolis, and has therefore to provide for a greater number of accidents than probably any other hospital of its size. All such cases are immediately admitted without delay or difficulty. About 25,500 patients were relieved during the past year, more than two-thirds of which were cases of accident or emergency. The Council earnestly appeal for donations and annual subscriptions. The present debt amounts to £11,000, and there are no investments which can be realised to relieve the hospital of this heavy burden. Secretary, Mr. A. E. Reade; Matron, Miss H. Gordon.

**German Hospital,** Dalston, N.E.—This hospital contains 125 beds, and admits all who are conversant with the German language, without distinction of nationality or creed, as well as accident and emergency cases. During last year 1,446 in-patients and 17,418 out-patients were treated at the Hospital Dispensary, and a further 6,004 at the Eastern and Western Dispensaries. Six beds are set aside for patients paying one and a half to two guineas a week. The convalescent home, with 17 beds, also has proved most beneficial. The annual expenditure amounts to about £9,800, the more reliable income to £5,100, leaving the sum of £4,700 to be raised by special appeal. Superintendent and Secretary, Mr. H. Gülich; Matron, Miss Christiane Bürger.

**Great Northern Central Hospital,** Holloway Road, N.—This hospital is entirely free to the sick poor, no letters of recommendation being required to gain admission. It was founded at King's Cross in 1856, but was removed to Holloway in 1887. The new hospital contains 155 beds, and was completed in 1894. It is one of the most beautiful and perfect in London, and every detail has been carried out in accordance with modern scientific knowledge. Over 1,300 in-patients and 26,000 out-patients are treated annually, but the increased number of beds now available will nearly double the number of in-patients. The hospital is unendowed, and the reliable income quite inadequate to meet the necessary expenditure—over £8,000 being required annually from voluntary sources. There is also a debt of £12,000 on the building fund—a heavy burden hampering the good work of the hospital. Annual subscriptions and donations may be sent to the Secretary, Mr. Lewis H. Glenton-Kerr.

**Guy's Hospital,** Borough, S.E.—Guy's is too old a friend of the public to need much to be said in its favour; but so long as it has closed wards and is crippled by poverty it is areproach to the metropolis. The net income of the hospital derived from its landed estates remains at £20,000 per annum less than it was before the agricultural depression, with no present prospect of improvement. There is left, therefore, only an assured income of £21,000 to maintain the 500 beds now open, which cannot be kept up at a less cost than £38,000 per annum. The greater part of this deficiency can only be met in the future as in the past, by liberal contributions from the public exclusive of any further aid from the same source, required to reopen 100 beds still remaining vacant, for which there is a pressing demand. Matron, Miss Nott Bower; Superintendent, Dr. Perry.

**King's College Hospital,** Portugal Street, W.C.—That this hospital has much claim on the public for the good work it does goes without saying. Its finances are not, however, in that flourishing condition that they ought to be in, were the hospital supported as it should be. First, there is a need for money to meet the current expenses, as of assured income it has but £1,000 to meet an expenditure of £19,000. Next comes the need of subscriptions towards paying off the loan due to the bankers; and thirdly, towards a Special Five Years' Maintenance Fund of £50,000, which was started by a meeting held at Grosvenor House under the presidency of His Grace the Duke of Westminster, K.G. The sum at present given and promised amounts to about £10,000. The average annual receipts of the hospital during the past ten years have been about £16,500, including legacies, but the annual expenditure is over £19,000. We must draw special attention to the convalescent home, which secures a period of ease for the patients sent to it from the hospital wards. As considerably over 25,000 patients are yearly admitted to the benefits of the hospital, it should surely have brought its claims for aid to the doors of many of the giving public, who, now knowing its needs, will, we feel confident, do their utmost to assist it. Secretary, Rev. N. Bromley; Sister-Matron, Miss Monk.

**London Hospital,** Whitechapel Road, E.—This is the greatest hospital in this country. From this fact, and from the excellent manner in which the work is carried on there, we make no doubt that the public will give largely to its funds, in testimony of their appreciation of the enormous value of what is being done there for the poor of East London. It includes special departments under eminent medical men for the treatment of all classes of disease, the number of beds devoted to children being greater than those to be met with in most children's hospitals. The character of the work done and its value to the public may be realised from the statement that the able matron, Miss Lückes, has four assistants under her, in addition to two night superintendents, 20 day sisters, and 220 staff and probationer nurses. This great hospital of the East-end is in serious want of funds, as the committee depend on voluntary contributions for £40,000 a year to enable them to maintain the 650 beds which are daily occupied by urgent cases. House Governor and Secretary, Mr. G. Q. Roberts; Matron, Miss Eva C. Lückes. Bankers: Messrs. Robarts, Lubbock, and Co., 15, Lombard Street, E.C.; Messrs. Glyn, Mills, Currie, and Co., 67, Lombard Street, E.C.

**London Homœopathic Hospital,** Great Ormond Street, W.C.—This hospital has a well-earned reputation for efficiency and careful management. The past year has witnessed the opening of the noble new building, equipped with all the latest sanitary and surgical requirements, and opened by the Duchess of Teck in July last, when the committee had the satisfaction of announcing that of a total cost of £45,000, £39,000 had been already given, leaving £6,000 to be collected to free the institution from debt. Since then a further £2,000 has been raised towards this desirable object, and an urgent appeal is being made for the remaining £4 000 in order that the hospital may start the new year entirely free from debt. To maintain the present hospital means an additional annual expenditure of £2,000 a year, and to relieve the committee of all anxiety of being able to keep open the 100 beds at their disposal, it is imperative that new annual subscriptions for this amount should be forthcoming. The treasurer is Viscount Emlyn, 7, Princes Gardens, S.W., and donations or new annual subscriptions will be thankfully received by him or by Mr. G. A. Cross, the secretary-superintendent, if sent to him at the hospital.

**Metropolitan Hospital,** Kingsland Road, N.E.—Situated in the heart of the poor and densely-populated districts of Shoreditch, Haggerston, Hackney, Bethnal Green, Hoxton, Dalston, and De Beauvoir Town, the hospital receives a very large number of accidents and casualities of all kinds, and the demands made on its resources, medical and surgical, are incessant and urgent. That the hospital is needed in its present position is shown by the fact that both the in and out patients have been steadily on the increase for several years past, the numbers treated last year being, in-patients, 781; out-patients' attendances, 78,238. In spite of this good work the expenditure last year exceeded the income by £1,346, leaving a debt of over £5,000 incurred during the past three years. It is deplorable that the work of the charity should be crippled for lack of funds, owing to which several beds have had to be closed, leaving only 66 now available for in-patients, although there is accommodation for 160. Many urgent cases have therefore constantly to be refused admission, and unless money is immediately forthcoming it will be still necessary to still further reduce the number of beds. Secretary, Mr. C. H. Byers.

**The Middlesex Hospital,** Mortimer Street, W. —This hospital contains 321 beds; the in-patients for last year numbered 3,182, and the out-patients 38,122 (total, 41,304); the income from all sources, including legacies and an amount of £8,452 specially contributed towards the cost of building a new cancer wing, was £41,445, and the expenditure (including the investment of the £8,452), £43,430; deficiency, £1,995. The cancer wards are a distinguishing feature of the hospital, and their extra nursing, costly treatment, and unlimited dietary add largely to the expenses of the hospital. A scheme, which has received the approbation of H.R.H. the Prince of Wales, is on foot to extend this department by removing it from the main body of the hospital and building a new wing, at a cost of £12,000, so £4,000 is still needed. A convalescent home containing 45 beds and isolation wards is in course of erection at Clacton-on-Sea. Secretary Superintendent, Mr. F. Clare Melhado; Lady Superintendent, Miss Thorold.

**North-West London Hospital,** Kentish Town Road, was founded in 1878, and is the only institution of the kind in the north-west district. It has 53 beds, of which 18 are for sick children. Last year there were 512 in-patients and 44,506 attendances as out-patients. The committee cordially invite visitors to visit the hospital to see and judge for themselves. There is also a Samaritan Fund, and a soup kitchen (maintained by the Treasurer, Mr. George Herring), where about 2,000 dinners weekly are being distributed. The annual expenditure is never under £4,000, towards which the annual subscriptions yield less than £900. The committee very earnestly plead for assistance to meet a present deficiency of £2,000. Secretary, Mr. Alfred Craske.

**Poplar Hospital for Accidents.**—The recent extensions at this hospital have greatly increased the work, consequently further subscriptions and donations are very necessary. The great benefit to the district and the energetic management of the institution should command hearty support. Secretary, Lieut.-Colonel Feneran.

**Royal Free Hospital,** Gray's Inn Road, W.C.—About £2,000 is now required. From the ruinous condition of the old front building, which was formerly a barrack for the Light Horse Volunteers, it became absolutely necessary to rebuild this portion of the hospital. This work has provided much-needed isolation wards, casualty-rooms, and dispensary, and a new steam laundry; and the committee solicit help towards supplying the amount now needed, also to increase the revenue by new subscriptions. The Royal Free Hospital was the first general hospital in London to open its doors freely to the sick and suffering poor without letters of recommendation from the governors. For 68 years it has adhered to this excellent system, and during that period over two and a quarter millions of poor people have been received within its walls. Secretary, Mr. Conrad W. Thies.

**St. George's Hospital,** Hyde Park Corner, S.W.—Occupying a commanding position, there is reason to believe that it suffers in consequence, and we hope that all who pass St. George's Hospital in future will contribute something to its funds in the course of the year. Secretary and Superintendent, Mr. C. L. Todd; Matron, Mrs. Coster.

**St. Luke's Hospital,** London, E.C.—This hospital continues its work of perfecting its building and adding to the comfort of its inmates. The expenses are necessarily very heavy, and the income quite inadequate to meet them. The committee are encouraged to confidently appeal for help to this one of the oldest of the London charities. Treasurer, Mr. Edward W. Nix; Secretary, Percy de Bathe, M.A.

**St. Mary's Hospital,** Paddington, W.—Very heavy work is thrown upon this institution owing to the large area of scattered poor which it has to assist. It is not so well supported as it should be, in spite of its many wealthy neighbours. The board of management, therefore, urgently appeal for further support. The hospital is free, and no urgent case is refused admission. Secretary, Mr. Thomas Ryan.

**St. Thomas's Hospital,** Westminster Bridge Road, S.E.—Early in the present year a special appeal was issued with the object of raising a sum of £100,000 to enable the wards now closed for lack of funds to be opened. Two of these wards are now available for patients, but £80,000 is still needed to make up this Sustentation fund. Subscriptions to Mr. Wainwright, the Treasurer, at the hospital.

**Seamen's Hospital Society, "Dreadnought,"** Greenwich, S.E.—This great national maritime hospital was established in the early part of the century. It has now relieved over 355,000 sick, injured, and shipwrecked sailors, and there are now more than double the number of seamen treated annually than as compared with ten years ago. Seamen come to the hospitals from every nation under the sun. At the "Dreadnought" (at Greenwich) over 8,000, and at the branch hospital, in the Victoria and Albert Docks, over 5,000 patients are annually cared for. There are also two dispensaries, in the East India Dock Road and at Gravesend. "All the world ought to subscribe for all the world send patients."—*Graphic*. Secretary, Mr. P. Michelli.

**University College Hospital,** Gower Street, W.—The North London or University College Hospital is mainly dependent upon voluntary contributions, the income on which it can rely being only £6,000, whilst the necessary annual expenditure is very nearly £20,000, and the present debt exceeds £11,000. The rebuilding of the hospital, too, upon a more modern plan, as soon as possible, is an urgent necessity, and for this purpose a fund was instituted in 1884, named the "Jubilee Endowment and Building" Fund, to which contributions are earnestly invited. Secretary, Mr. Newton H. Nixon.

**West London Hospital,** Hammersmith Road, W. (101 beds), is the nearest for a population of nearly 500,000 persons. The accommodation for in-patients is lamentably short of the number requiring admission, and in each succeeding year the fact is brought more to the front than in its predecessor. Last year (1894), the beds were occupied by 1,522 patients, and the out-patient department was attended by 31,933 others. Its endowment yields an annual income of only £115. The Board appeal for funds for continuing the erection of a section to accommodate about 80 beds, and for carrying on the present work. Secretary, Mr. R. J. Gilbert; Lady Superintendent, Miss Hardy.

**Westminster Hospital,** Broad Sanctuary, S.W.—Out of an expenditure of £15,000, less than £3,000 is assured to this charity, so that about £12,000 has to be made up each year in subscriptions. The hospital does an immense service to the country by training a large number of excellent nurses. Secretary, Mr. Sidney M. Quennell; Matron, Miss Pyne.

### PROVINCIAL HOSPITAL.

**The Birmingham General Hospital.**—This usefu institution is continuing its work under difficulties until the completion of the fine building in process of erection. Funds are most earnestly appealed for towards the building fund and general maintenance of the hospital. The expenditure far exceeds the assured income, and annual subscriptions are especially needed. House Governor, Mr. Howard Collins.

### SPECIAL HOSPITALS.

### CONSUMPTION.

**Brompton Hospital for Consumption and Diseases of the Chest,** S.W.—This hospital is well known as one where the patients are well cared for, and every effort is made to brighten their clouded lives. Many subscriptions have been withdrawn through failure of income and other causes; the committee are most anxious that their places should be filled up by other kind friends. Secretary, Mr. W. H. Theobald.

**City of London Hospital for Diseases of the Chest,** Victoria Park, E.—This hospital is situate in the East of London, where the diseases it treats are so common. About 1,000 in-patients and 15,000 out-patients are treated yearly. The average annual expenditure is more than £11,000, towards which only £370 of the income is assured. Assistance is much required. Donations or annual subscriptions will be thankfully received by the Secretary, Mr. T. Storrar-Smith; or by the bankers, Messrs. Barclay, Ransom, and Co.

**North London Hospital for Consumption,** Mount Vernon, Hampstead, N.W., and 41, Fitzroy Square, W.—This institution was thoroughly reorganised and remodelled some years ago, and has now become one of the most useful of its class; 400 in- and 3,000 out-patients are admitted each year. Secretary, Mr. W. G. Farrance Bosworth. Matron, Miss K. Elphick.

**Royal Hospital for Diseases of the Chest,** City Road, E.C.—This is the oldest consumption hospital in Europe, having been founded by Her Majesty's father, the late Duke of Kent, in 1814. There are 80 beds, and last year 528 in-patients and 7,739 out-patients were treated. The expenditure exceeds £7,000, towards which there is an annual subscription list of £2,000, and dividends amounting to about £100. Two additional wards have recently been opened, and funds are urgently needed. Donations will be gratefully acknowledged by the Secretary, Mr. John Harrold, or may be sent direct to the Chairman of the hospital, Mr. T. Andros de la Rue.

**The Royal National Hospital for Consumption and Diseases of the Chest at Ventnor,** Isle of Wight, provides accommodation (including separate bedrooms) for 134 patients (83 men and 51 women). Every bed is occupied, and many candidates are waiting for admission. As the expenses exceed the assured income by £4,000, the committee appeal for additional annual subscriptions and donations. The bankers to the charity are the London and Westminster, St. James' Square, S.W., and the secretary, Mr. Ernest Morgan, will gladly furnish further particulars on application at the London office, 34, Craven Street, Charing Cross, W.C.

### LYING-IN.

**The City of London Lying-in Hospital,** City Road, E.C.—The number of patients at this hospital again shows an increase, over 2,200 poor women having been safely delivered either in the hospital or at their own homes, only two maternal deaths having taken place in the hospital since May, 1891. This fact is a sufficient proof that the antiseptic treatment is fully carried out. The committee have recently enlarged the hospital at a cost of over £2,000; and earnestly appeal to the public for additional support to meet this outlay, and to enable them to carry on the general work of the hospital. Secretary, Mr. R. A. Owthwaite.

### EPILEPSY AND PARALYSIS.

**Hospital for Epilepsy and Paralysis, and other Diseases of the Nervous System,** Regent's Park.—Nowhere, perhaps, can there be found a more distressing and incapacitating class of diseases than those, the sufferers from which seek relief at this hospital. In such an institution as this members of the profession find that wide experience which alone can enable them to combat these diseases and to successfully treat them. It is to public sympathy and liberality that this hospital appeals for £1,000 to enable it to fulfil its task. Secretary, Mr. H. Howgrave Graham.

**National Hospital for the Paralysed and Epileptic** (Albany Memorial), Queen Square, W.C.—There are 180 beds at this hospital, a number painfully insufficient to meet the requirements, as in a large majority of cases the patients are unsuited to general hospitals. The attendances of out-patients are upwards of 30,000 yearly. More than 1,500 different cities, towns, and villages have sent in patients. Besides the hospital for treatment, there is a pension fund for the incurable. Annual expenditure is nearly £15,000, of which more than £8,000 must be raised in benefactions. A new country and convalescent home is in course of erection, which when finished will raise the total number of beds to nearly 200. Director, Mr. B. Burford Rawlings; Matron, Miss L. C. East.

**West-end Hospital for Paralysis, Epilepsy, &c.,** 73, Welbeck Street, W.—A special appeal is now being made to enable the committee of this institution to pay off a debt of £6,000 incurred in rebuilding. The hospital contains fifty beds. Treasurer, Mr. H. Alexander Dowell.

### CHILDREN.

**East London Hospital for Children and Dispensary for Women,** Shadwell, E.—If institutions such as St. George's and St. Mary's Hospital suffer from absence of adequate support, it will readily be understood that the struggle for existence is much greater in such a neighbourhood as Shadwell. The committee appeal to the public to enable them to complete most necessary improvements, costing £2,500, besides earnestly asking for further subscriptions and donations also. Mr. Thomas Hayes, Secretary.

**North-Eastern Hospital for Children,** Hackney Road, Shoreditch.—This hospital, placed in the midst of one of the most crowded districts of East London, does an excellent work amongst the children of the poor. Its power for usefulness is much crippled for lack of beds, an extension of its premises being sadly needed, but before this can be accomplished a debt of £3,500 remains to be paid off. To do this, and to raise the income of the hospital by £500 a-year, a special appeal has lately been issued, and subscriptions and donations are earnestly asked for. Secretary, Mr. T. Glenton-Kerr; Matron, Miss E. W. Curno; City office, 27, Clement's Lane, E.C.; Bankers, Barclay, Bevan, and Co.

**The Hospital for Sick Children,** Great Ormond Street, London, W.C.—Founded in 1852, with ten beds, this was the first hospital solely devoted to the sick children of the poor. There are now nearly 200 beds at Great Ormond Street, besides 52 beds at Highgate. About 1,700 in-patients, besides about 22,000 new out-patients, have be n treated in 1894. £1,000 asked for in new annual subscriptions to meet cost of maintenance. £3,000 required to free the hospital from the burden of debt on the new building. Secretary, Mr. Adrian Hope; Lady Superintendent, Miss Smedley.

**Victoria Hospital for Children,** Queen's Road, Chelsea, and Victoria Convalescent Home, Broadstairs.—Established at Chelsea in 1866 for the relief of the sick and suffering children of the poor, this unendowed hospital seeks further subscriptions to help it to carry on its work. The expenditure, which is only 19s. per patient, yet reaches £7,000 to £8,000 a year, and this has to be raised entirely by voluntary subscriptions. Secretary, Commander W. C. Blount, R.N.; Matron, Miss Hamilton.

### WOMEN.

**Chelsea Hospital for Women,** Fulham Road, S.W.—This hospital has 52 beds, and treats respectable poor women and ladies in reduced circumstances. Contributing in-patients pay 10s. 6d. to 42s. a week, according to their means. The hospital is entirely without endowment or reserve funds, and earnestly desires more annual subscriptions. Secretary, Mr. Herbert H. Jennings; Matron, Miss Mildred Heather-Bigg.

**Grosvenor Hospital for Women and Children,** Vincent Square, Westminster, S.W.—The Grosvenor Hospital is situated in a densely-populated district of Westminster, and the bulk of the out-patients is drawn from the poorer classes living in the neighbourhood. In-patients, however, are received from all parts of the country, and both residents and non-residents of London may well give the institution a generous support. The hospital has been lately enlarged to meet the increased demands for its benefits, and the current annual expenditure is consequently increased. Secretary, Mr. A. S. Harvey; Matron, Miss K. Hughes.

**Samaritan Free Hospital for Women and Children,** Marylebone Road, N.W.—Some £7,000 per annum is required to maintain this hospital, of which only £1,750 can be relied upon in annual subscriptions. The committee would therefore like to see a great addition to the list of annual and life subscriptions, and earnestly appeal for Christmas and New Year's gifts. Bankers, Scott and Co., 1, Cavendish Square, W. Secretary, Mr. G. Scudamore; Matron, Miss Butler.

**The Hospital for Women,** Soho Square, W.—This institution claims the distinction of being the first established of its specialty, having been founded in the year 1842. There are 60 beds in constant use, and funds for their maintenance are much needed. The committee very earnestly appeal for additional annual subscriptions. Secretary, Mr. David Cannon; Matron, Miss Squier.

**The Royal Hospital for Children and Women,** Waterloo Bridge Road, S.E.—This hospital stands in the midst of the densest poverty—indeed, with the single exception of St. George's in the East, the population is more crowded than that of any other portion of London, being 216 persons to the acre. This year the demand on the beds has been exceptionally high; the income of the year is insufficient to enable the hospital to continue to keep open all the wards unless further help is obtained. A sum of £1,000 is wanted, and that immediately, if this valuable institution is adequately to perform its good work. Secretary, Mr. G. H. Southern; Matron, Miss Beatrice F. Hunter.

### MISCELLANEOUS—SPECIAL.

**Central London Ophthalmic Hospital,** Gray's Inn Road, W.C.—This hospital, which has treated 267,323 patients during the fifty-two years of its existence, is in much need of funds. Secretary, Mr. William Abrams.

**Dental Hospital of London,** Leicester Square.—The Dental Hospital is contemplating moving to a new site as soon as the money required is forthcoming, the sanitation and accommodation of the present building being inadequate. Dentistry is the one branch of medicine that is justified in having a special hospital, and the committee can assure the benevolent public that the Dental Hospital of London is in every respect worthy of support. The bankers are Messrs. Barclay, Ransom, and Co.; the Treasurer, Mr. Joseph Walker, M.D.; and the Secretary is Mr. J. Francis Pink.

**Lock Hospital and Home,** Harrow Road, W.—Rescue work, as well as the cure of the patients who present themselves for admission, is the aim of this institution, and the good work has only to be known to meet with the support it deserves. A considerable number of those that pass through the hospital are reclaimed from their evil lives, which is a great result. A debt of under £500 has to be cleared off, and £1,000 is much required in fresh annual subscriptions to meet the regular requirements of the institution, and prevent the recurrence of debt. Secretary, Mr. A. W. Cruikshank; Matron of Hospital, Mrs. Kydd; Matron of Home, Miss J. McNiven.

**London Fever Hospital,** Liverpool Road, Islington, N.—The resources of this hospital have been much taxed during the last two years owing to the prevalence of scarlet fever. The institution is dependent upon voluntary support, and donations and subscriptions will be gratefully received. Secretary, Major W. Christie.

**Royal London Ophthalmic Hospital,** Moorfields, E.C.—An urgent appeal is made by the Board of Management for funds in support of this institution. The expenditure amounts annually to over £6,000 whereas the certain income is about £2,000. The patients numbered in 1894, in-patients, 1,981; out-patients, 113,555. All patients are admitted free, and without letters of recommendation. Bankers: Williams, Deacon, and Co., 20, Birchin Lane, E.C.; Secretary, Mr. Robert J. Newstead; Matron, Miss Robinson.

**The National Orthopædic Hospital,** Great Portland Street, seeks to restore the deformed to natural shape, and give new strength and vitality to those who are handicapped in the struggle for existence. The process of making the crooked straight and of setting the captives free is most often long and tedious, and the number of patients cannot therefore be compared with more general institutions. The hospital's pressing needs are first to see the end of a debt of £1,650 on the building, and to increase its list of annual subscribers to three times its present length. The beds are always occupied, and about thirty patients accepted by the surgeons wait for admission on the occurrence of vacancies. Secretary, Mr. H. J. Tresidder.

**Western Ophthalmic Hospital,** 155, Marylebone Road, W.—At present existing in a private house, quite unfitted for hospital purposes, and growing needs, the necessity for erecting new buildings has become urgent, and Sir Reginald Hanson, treasurer to the hospital, has issued an appeal for funds towards this desirable end. Help is earnestly asked for.

### A FEW OTHER CHARITIES.

**Association for the Oral Instruction of the Deaf and Dumb.**—The association has publicly introduced into the United Kingdom the German or pure oral system for teaching deaf and so-called dumb children to speak *viva voce*, and to understand the spoken words of others by lip-reading. Its offices are at 11, Fitzroy Square, W. The association maintains a training college for teachers of the deaf (both men and women) and a normal school for deaf children of all classes and denominations, and has issued many publications. The expenses of the association are very heavy. They are met by voluntary contributions and fees, which fall short of what is needed by about £700 per annum, and at the present moment the association is in debt to its bankers to the amount of £2,500. Financial aid is most earnestly solicited, and cheques may be directed to the Secretary, 11, Fitzroy Square, W.

**Bethnal Green Free Library.**—This pioneer institution, which is freely open to all comers, has now been in existence nearly twenty years, and has just commenced its winter's work, viz., evening classes for technical instruction to the youth of both sexes at reduced fees. The chief feature of the establishment is, of course, the library, which is the third largest in London, containing between 30,000 and 40,000 volumes. The extreme poverty of the people in the surrounding neighbourhood compels the committee to make an urgent appeal for funds to meet outstanding liabilities. Contributions will be thankfully received by the Librarian, at the Bethnal Green Free Library, E.

**Christian Men's Union Gospel Mission (Unsectarian),** 64, Chalton Street, Somers Town, N.W. —This mission, which devotes itself to the alleviation of the very poor in the region of St. Pancras and Somers Town, appeals for help towards providing dinners on Christmas Day for six hundred little children, and coals and other necessaries for five hundred of the most destitute and deserving old people in the district. Treasurer, Mr. Herbert Page, 490, Caledonian Road, N.

**City of London Truss Society,** 35, Finsbury Square.—For the relief of the ruptured poor throughout the kingdom. At the present time about 10,000 of both sexes and all ages are treated annually, while over half a million patients have been relieved since its formation eighty-eight years ago. The recently enlarged premises provide separate entrances for male and female patients; and there is a female attendant for the latter. Secretary, Mr. John Whittington. N.B.—Since the separate entrance has been provided for them, the increase in the number of female patients has been considerable; and should lead to an increased number of lady subscribers to the funds of the charity in aid of their poorer sisters.

**Hospitals for Women in India.**—The Zenana Bible and Medical Mission has now three hospitals doing most useful work amongst the women of India, at Lucknow, Benares, and Patna. They carry on an extensive work, besides educational, medical, and religious, amongst the female population of India, and are in need of constant help to continue and extend their labour. General Secretary, the Rev. A. Cavelier; Hon. Secretary, Mr. W. T. Paton. Offices: 2, Adelphi Terrace, W.C.

**London Orphan Asylum, Watford.**—Five hundred children are now profiting by the advantages given through this old-established charity, no less than one hundred having been admitted during the past year. Donations towards the necessary income, entirely derived from voluntary sources, will be thankfully received by the Secretary, Mr. H. C. Armiger, 21, Great St. Helen's, E.C.

**Mary Wardell Convalescent Home for Scarlet Fever,** Stanmore, Middlesex.—The only home for convalescents from scarlet fever. The demand on its forty beds is great, and expenses heavy. One thousand pounds urgently needed at the present time to help pay off debt and meet incoming bills. Subscriptions to Miss Mary Wardell.

**Metropolitan Drinking Fountain and Cattle Trough Association.**—This is the only society which provides free supplies of water for the many thousands of men, women, and children, besides horses, cattle, sheep, and dogs that are daily toiling through our streets. It depends entirely on voluntary contributions, which are urgently needed. Secretary, Mr. M. W. Milton, 70, Victoria Street, Westminster, S.W.

**Orphan Working School,** Haverstock Hill and Hornsey. Offices, 73, Cheapside, E.C. Founded 1758.—This is a national and undenominational institution which maintains 500 children, varying in age from infancy to fourteen or (in special cases) fifteen years. It is greatly in want of funds at the present time, urgently needed to meet immediate and pressing liabilities. This orphanage is the oldest one of the kind in the metropolis, and a large percentage of scholars turn out satisfactorily. Secretary, Mr. Algernon C. P. Coote, M.A.

**Royal Albert Orphan Asylum,** Bagshot.—This institution affords a home and industrial training for about 200 fatherless children. No canvassing or sale of votes is permitted. A full list of applicants, with the circumstances of each case fully detailed, is submitted to subscribers, who are thus enabled to help the most needful cases. Help is urgently needed, as the expenditure has exceeded the income, and a curtailment of the work will be necessary unless funds are forthcoming. Bankers, Lloyds Bank, 72, Lombard Street. Secretary, Mr. H. W. Tatum.

**Royal Sea Bathing Infirmary for Scrofula,** founded at Margate 1791.—This unique and valuable institution is so badly supported that many of the fine wards have been reluctantly closed. It is the only hospital in England set apart exclusively for the treatment of scrofulous disease, and the cures which result from a lengthened stay are indeed remarkable. We are glad to hear that wards containing ten beds have been reopened during this year. All information will be gladly given by the Secretary, 30, Charing Cross. Treasurer, Mr. M. Biddulph, M.P.

**St. Giles' Christian Mission,** 4, Ampton Street, Regent Square, W.C.—Doing all that can be done for the immediate and pressing needs of the starving and friendless in the squalid courts and alleys of St. Giles, meeting discharged prisoners, and holding out a helping hand in a time of sore distress and hopelessness, the unwearied workers of this mission are sorely crippled in their labour of love by lack of funds. Let those who cannot give personal service for this cause spare what they can, even the smallest sums, and forward to Mr. Wheatley, Superintendent, at above address.

**Thames Church Mission,** 31, New Bridge Street, E.C.—Most people have a warm corner in their hearts for sailors and seamen generally. Money to carry on its helpful work amongst the seafaring comers and goers on the Thames is much needed by this society. Secretary, Mr. F. Penfold.

**The Surgical Aid Society,** Salisbury Square, E.C. —This society has made steady progress, financially, and in the sphere of its work. The annual subscriptions have reached the sum of £6,571, and the total income last year was £10,592, and no less than 87½ per cent. of this amount has been expended in actual relief. During the past year the large total of 20,046 appliances were given away. There is ample scope for very considerable extension of these benefits, and therefore the committee earnestly appeal for contributions. Secretary, Mr. Richard C. Tresidder, to whom subscriptions should be made payable.

**The Morley Convalescent Home for Working Men.**—This institution has been in existence for over thirteen years, the progress and development made being of a remarkable character. Starting with an accommodation of twenty-four beds, eighty-six beds are now provided. It was established by a few London working men. The management of the institution appeal to the sympathy and support of all who are desirous of furthering the interests of this beneficent movement by contributing towards the £400 necessary to erect a new wing now urgently needed. Hon. Secretary, Mr. W. Goad.

**The Church of England Scripture Readers' Association.**—The society employs 134 readers who, visiting amongst the very poorest of the population, have much opportunity for doing good. A special effort is necessary to remove the deficiency of £4,014 on the accounts of the society, and thus enable it to continue the work. Subscriptions and donations will be gratefully received by the Secretary, Miss M. Tilby. Offices, 56, Haymarket, S.W.

### Special Christmas Appeal for the Hospitals.

"He gives twice who gets a friend to give."—*The Hospital.*

☞ THE BANK ORDER GIVEN BELOW SHOULD BE SENT ENCLOSED IN AN ENVELOPE ADDRESSED TO THE SECRETARY OF THE SELECTED INSTITUTION.

### Bank Order.

December, 1895.

To ____________________________
Bankers.

## Figures and Tables

**Figure f1:**
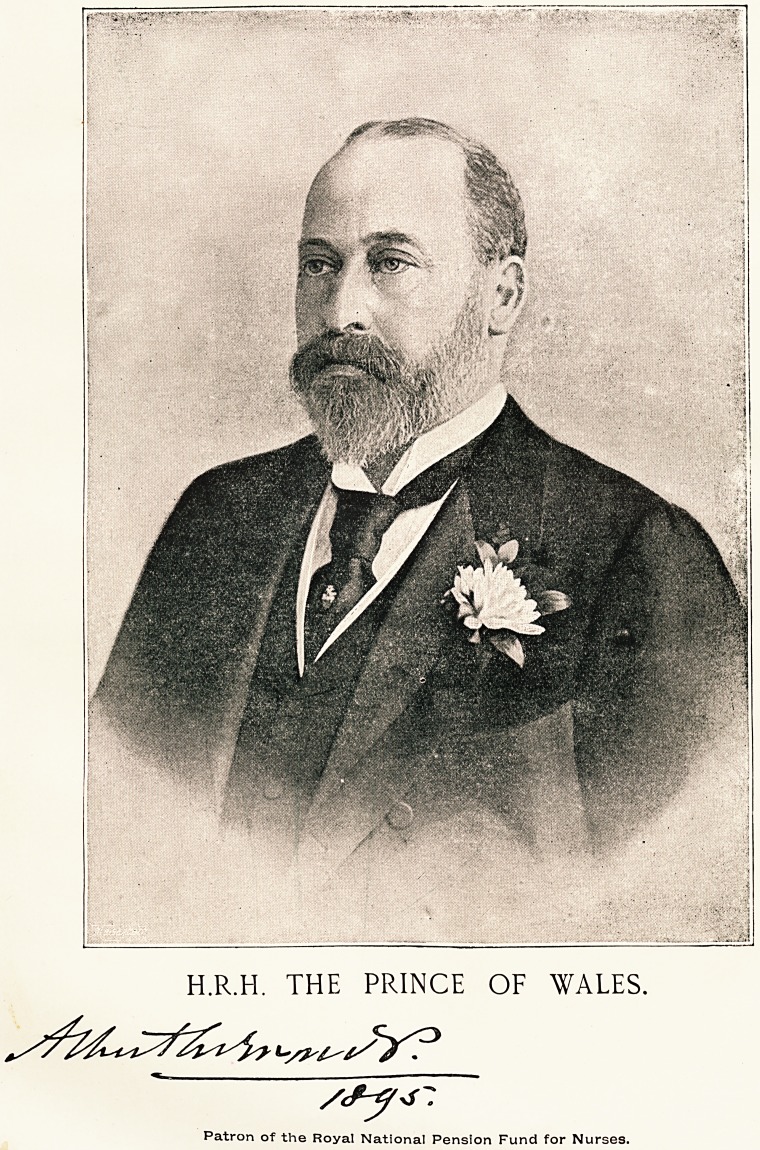


**Figure f2:**
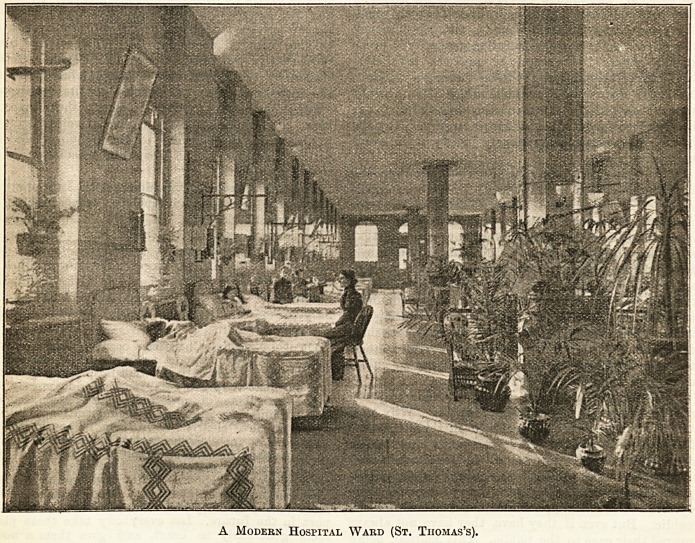


**Figure f3:**
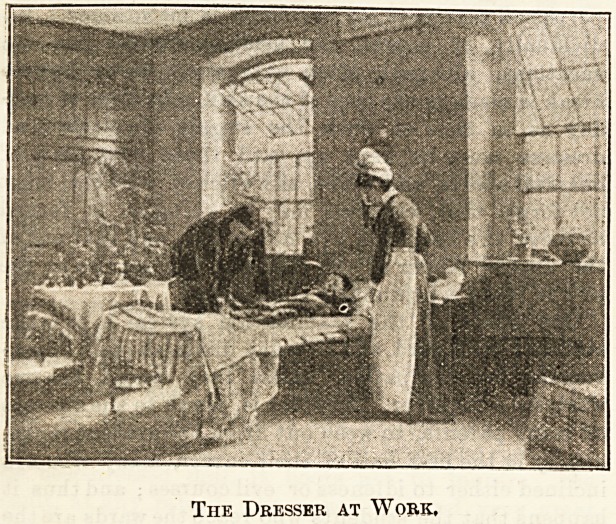


**Figure f4:**
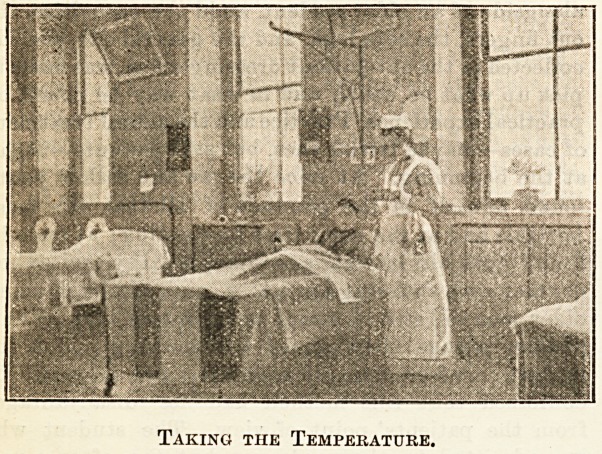


**Figure f5:**